# Discourse on Intellectual Disability and Improved Access to Assistive Technologies in Malawi

**DOI:** 10.3389/fpubh.2018.00377

**Published:** 2019-01-29

**Authors:** Peter Morris Gasten Ngomwa

**Affiliations:** Malawi Council for the Handicapped, Limbe, Malawi

**Keywords:** intellectual disability (ID), discrimination, sensory impairment, Malawi, disability act, equality, health services

## Abstract

Assistive technologies are one of the five elements under the Health Component of the World Health Organization CBR Guidelines that Malawi is using to implement the Community Based Inclusive Development (CBID) Programme. The technologies enhance independent living by removing barriers that come due to disability or old age and should, therefore, be prioritized. However, Malawi does not have a straightforward way of providing Assistive Technology. Individuals are considered upon the assessment of their needs whose intervention with respect to assistive products may not be available. This is mostly the case with persons with intellectual disabilities, in which there is very little expertise to work with, in Malawi, although they require assistive products to improve their quality of life just like other persons with disabilities. There are many sectoral policies and laws in Malawi, nonetheless, they do not have a positive input on persons with intellectual disability to access assistive technologies in terms of availability (provision), affordability (cost), and appropriateness (suitability and quality). Therefore, this paper intends to demonstrate the barriers that are faced by persons with intellectual disabilities, examine the policies, and pieces of legislation that would have influenced better access and maps the way on how barriers can be removed to ensure that Assistive Technologies are readily and easily accessed.

## Introduction

Persons with intellectual disability in Malawi are more disadvantaged and pushed to the margins of society. These may consist of mental illness, Down syndrome, autism asparagus syndrome, epilepsy, some types of cerebral palsy, and severe cases of hydrocephalus. Persons with intellectual disabilities, therefore require support because of the characteristics like slow learning, uncontrollable body movements, slurred speech, problems in decision making, and memory problems^1^. It is, therefore, very important that the government should initiate and improve services toward their inclusion. It includes access to appropriate technologies that are very essential for them to have equal opportunities like anyone else^2^.

The Community Based Inclusive Development (CBID)^3^ program that was introduced in 1988 as a pilot project in Blantyre district (1) and is now extended to half of the country has not made a noticeable response to the diversity of disability and needs. In general, most of the support is delivered to persons with mobility and other physical challenges, visual and to a limited extent persons with hearing impairments. As a result, insufficient consideration has been given to persons with intellectual difficulties and those with developmental impairments (2). Limitations include lack of responsiveness to diversity; priority to access medical, education, and livelihood interventions; most CBID models that were fragmented were not aligned to the Government development structures at the community, district and national level^4^. On top of these there were also other challenges like sustainability being depended on the goodwill of volunteers; limited coverage and dependence on donor funding.

**Figure 1:**
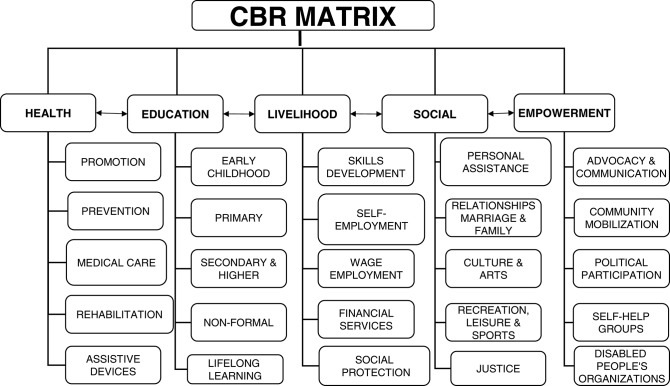
The World Health Organization CBR Matrix—Components and their elements (see as above).

However, Malawi has developed a new National Harmonized CBID Model (2) whose main features are diversification and inclusiveness; partnership and collaboration; capacity building; participation of persons with disabilities; ownership and sustainability by district councils; and compliance with national and international standards. The new model that has adopted the World Health Organization CBR Matrix^5^ also has a monitoring and evaluation framework targeting the district councils; has mechanisms for data collection and management, and is responsive to the local context (see Figure 1).

**Figure 2:**
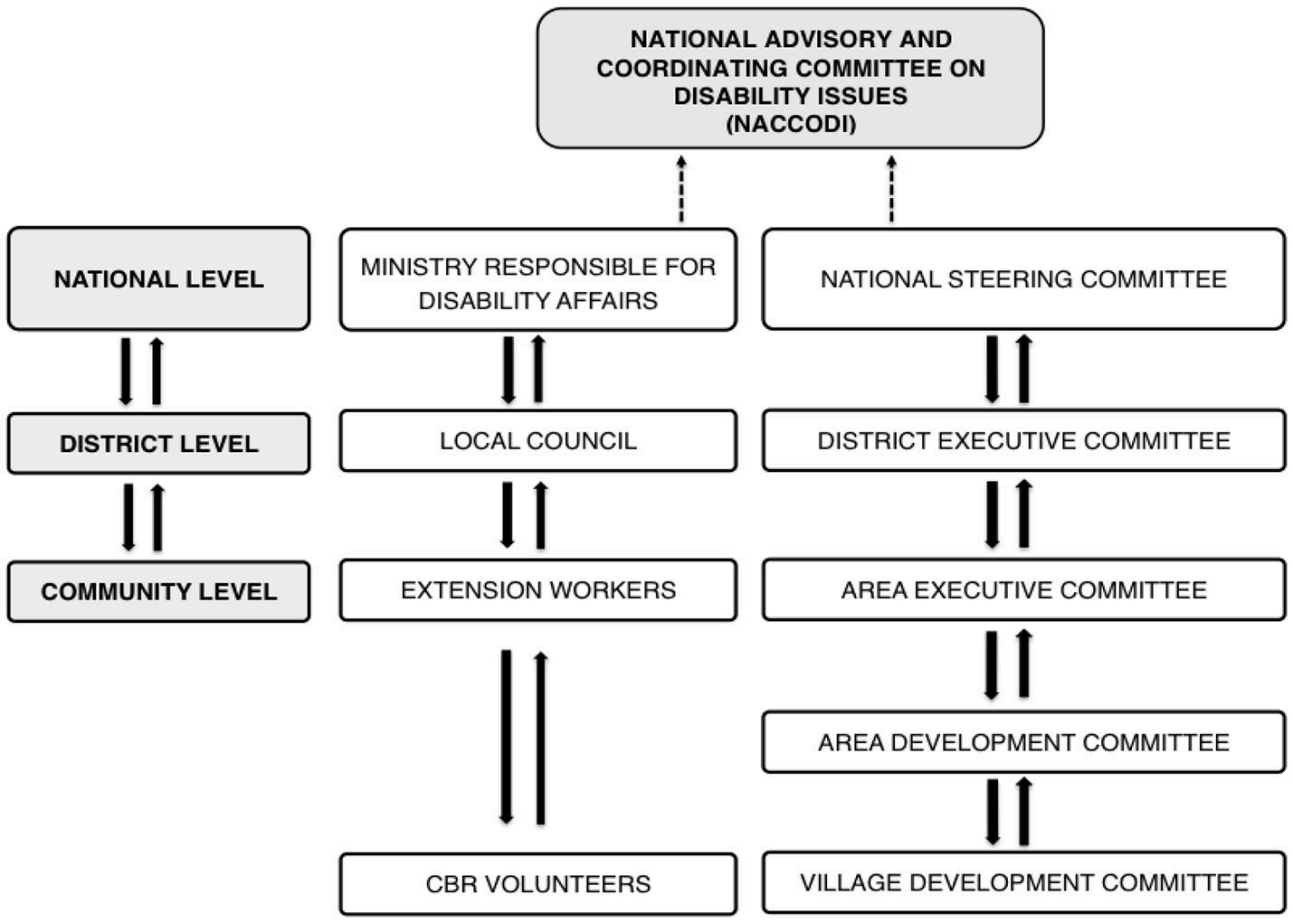
The Local Government Structure.

Since CBID is a rights-based approach to disability issues, it will ensure that persons with intellectual disabilities are included in all the intervention done under it comprising access to assistive technologies by persons with an intellectual impairment. This will be through identification of persons with intellectual disabilities, assessment and referrals to appropriate service providers depending on their needs using the CBID mechanism that uses the local Government structure^6^ and has volunteers who move door to door at the village level (see Figure 2).

The Malawi Disability Act, 2012, defines disability as a “long-term physical, mental, intellectual or sensory impairment, which in interaction with various barriers may hinder the full and effective participation of a person on an equal basis with other persons”^7^. Thus, the advancement of inclusive development by mainstreaming disability would be demonstrating an understanding that persons with intellectual disabilities have to enjoy human rights like other persons other than taking them as objects of charity. Since Malawi signed and ratified the United Nations Convention on the rights of Persons with Disabilities (CRPD) in 2007 and 2009, respectively^8^ there is need of policy, legal and systemic reforms to ensure that persons with intellectual disabilities access appropriate technologies in Malawi.

Therefore, section Situation Analysis of this paper highlights the challenges faced by persons with intellectual disabilities in Malawi. It includes negative perceptions of them and the lack of technical know-how available to this type of disability. Section Overview of the Legal and Policy Framework analyses the current disability legal instruments and policy frameworks in comparison to the international standards in the provision of assistive technologies and its implications on persons with intellectual disabilities. Section Recent Developments justifies why CBID is the best way forward to improve the situation, followed by section Actionable Recommendations and the paper concludes.

## Situation Analysis

Persons with disabilities in Malawi, just like in numerous other countries, have many challenges. These result in getting them marginalized from the mainstream society that makes it problematic for them to realize their essential social, political, and economic rights^9^. “The majority of them have a difficult life since they are poor, abandoned, uneducated, malnourished, discriminated, neglected, and vulnerable. The factors contributing to this pathetic situation are many and varied but include poverty, unemployment, and social isolation, environmental, institutional, attitudinal, and economic barriers” (as above).

### Misconceptions

Since persons with disabilities experience discrimination since birth or the moment they acquire a disability, life becomes a struggle from there onwards. It is also true with intellectual disability that is associated with some myth and misconceptions. An example that is given by Joseph Kisanji that talks about attitudes toward disability in Tanzania (3) would not be different to Malawi as a neighboring country. Joseph gives instances of proverbs used in Tanzania that show that intellectual disability is dreaded and not acceptable in the society (3). Persons with intellectual disabilities are believed to be a curse upon the family. Opinions like this make it difficult for individuals with intellectual disability to be taken as an equal citizenry. Because of such myths and misunderstandings, persons with intellectual disabilities are consequently severed and hidden from the public^10^.

### Lack of Enabling Environment, Mechanisms, and Resources

SINTEF^11^ conducted a study that found out that a significant part of the sample that was used in the study indicated that they had problems to access health services. For example, of the 84% of the sample that required health services merely 61% received it and only 5% accessed assistive devices out of the 69% who were in need of them^12^.

Usually, health services are to a certain extent not accessible to persons with intellectual disabilities due to the key challenges that include a lack of appropriate technologies, inappropriate laws and policies, and their subsequent reinforcement. In addition, severe gaps in human and financial capabilities in the production of assistive products are there because the Government has not decentralized this to the private sector. Decentralization to the private sector would have enhanced competition, thereby, improving production; affordability due to competitive prices; quality; and appropriateness.

An interview with the Acting Principal of Montfort Special Needs Education Centre revealed that the Centre for Learning Difficulties does not use any appropriate technology^13^. She claims that learners with an intellectual disability simply need more time to learn since that is their problem. However, in another interview with the Programs Manager of the Parents of Disabled Children Association in Malawi (PODCAM), it is learnt that appropriate technologies can be used to assist persons with intellectual disabilities with their learning^14^. The PODCAM official points out that children with intellectual disability require assistive technologies, for example, computer games to stimulate their minds. It may be attributed to lack or low demand for the devices due to exposure or donor support being restricted to some geographical area^15^ and includes lack of the CBID programme.

It is evidenced from neighboring countries like South Africa where they use Assistive Listening Devices (ALDs) that assist in amplification of sounds so that somebody is able to hear better; Augmentative and Alternative Communication (AAC) devices to assist individuals with communication impairments to express themselves; and Alerting Devices that assist those with hearing loss^16^. Hearing aids and sign language is also used for communication purposes^17^.

Technology assists persons with intellectual disabilities in many ways like communication cited above. But appropriate technology can also be used for mobility like using wheelchairs that are controlled by computers; environmental control where electrical appliances, video and audio equipment can be operated by devices. Appropriate technology is also used in activities of Daily Living (ADL) like feeding, assisting individuals with memory difficulties to accomplish an activity or instructional materials that are in video form. In Uganda toys and playing materials can also be modified to ease play by children with intellectual disabilities^18^. Nonetheless, most of these are not available in Malawi.

### Lack of Data

The absence of disability-specific data on persons with an intellectual disability makes it quite difficult to locate them and establish the need for assistive devices. It is the reason the majority is not reached. It, therefore, necessitates the issue of data collection and management which is one of the features of CBID.

## Overview of the Legal and Policy Framework

### International Law on Disability

#### The United Nations Convention on the Rights of Persons With Disabilities (CRPD) 2006

There are several international instruments but the most recent and specific to persons with disabilities is the United Nations Convention on the Rights of Persons with Disabilities^19^. It obligates States as in the following clauses:-

- **General Obligations**: promotion of research and development and provision of accessible information to persons with disabilities on appropriate technology and support services^20^.
- **General Mobility**: Facilitate access and encourage production of assistive technologies^21^.
- **Habilitation and Rehabilitation**: Promote the availability, knowledge, and use of assistive devices and technologies^22^.
- **Participation in Political and Public life**: Use of assistive technologies in the protection of the rights of persons with disabilities, for example, voting, health, education, employment, and community participation without barriers^23^.
- **International Cooperation**: Access, sharing, and transfer of assistive technologies^24^.

However, adequate domestication and enforcement are required to ensure compliance with the laid down international standards. Malawi has attempted to domesticate these obligations as highlighted below.

### The Local Legal Framework on Disability

#### The Malawi Republican Constitution, 1995

Section 20(1) of the Bill of Rights in Chapter IV of the Malawi Republican Constitution^25^ guarantees equal and effective protection for all persons and prohibits discrimination on any basis including on the basis of disability among others. Additionally, Section 13^26^ calls upon the State to progressively develop policies and legislation toward achieving the goals of children and persons with disabilities, gender equality, health, and education among others. For persons with disability, the principle in section 13(g)^27^ offers recognition of processes to the advantage of persons with intellectual disabilities to access and be actively involved in the community. Although not explicit, it would include the issue of assistive technologies that improve the quality of their lives.

#### The Disability Act 2012

Section 26(e) of the Disability Act says “promote the design, development, production, and distribution of accessible information and communication technologies and systems, and ensure that the same are available for persons with disabilities at an affordable cost”^28^. The provision, although laying emphasis on information and communication, when coupled with section 4.6.1.2 of the National Policy on Equalization of Opportunities for Persons with Disabilities^29^ guarantees issues of social, economic and participation of persons with intellectual disability through the provision of assistive technologies they require.

#### Education Act of 2013

The 2013 Act that is attached to the values of accessibility, quality, relevance, efficiency, equality, equity, liberalization, partnership, decentralization, transparency, and accountability as also proclaimed in the Republican Constitution promotes equal access to education for all people in Malawi. Since the values are accorded irrespective of race, ethnicity, gender, religion, disability, or any other discriminatory characteristics it would assist in removing the discriminatory tendencies to persons with intellectual disabilities to access the assistive terminologies they need^30^.

#### Handicapped Persons Act, Cap 33:02 of 1971

The Handicapped Persons Act of 1971^31^ is currently being reviewed as it was based on the charity model of disability. However, it gives powers to the Malawi Council for the Handicapped (MACOHA) to design, implement, and monitor rehabilitation programs and services for the socio-economic empowerment of persons with disabilities. MACOHA also regulates operations of organizations of and for persons with disabilities. It creates awareness on disability and facilitates active involvement of the community in disability issues.

MACOHA is thus well-placed, to make sure that persons with intellectual disabilities have access to assistive technologies through the planning of disability programmes and services in the country.

#### Employment and Labor Act

Section 5 of the Employment and labor Act of 2000^32^ which is the legal framework for regulating the basic employment conditions outlaws discrimination on the basis of disability whether at recruitment or during the course of employment or termination. It additionally encourages positive actions for the underprivileged groups, including persons with intellectual disabilities.

The Act thus gives another opportunity for non-discrimination of persons with intellectual disabilities to access assistive products or technologies in employment processes stipulated above.

### The Policy Framework

There are several sectoral policies that would assist in issues of access to appropriate technology although they do not explicitly mention intellectual disabilities. The policies have provisions that can be used to assist the development of assistive technologies and improve access by persons with intellectual disabilities. Some of the policies that are relevant enough are as follows.

#### National Policy on Equalization of Opportunities for Persons With Disabilities (NPEOPWD), 2006

The National Policy on Equalization of Opportunities for Persons with Disabilities^33^ has the purpose of promoting the rights of persons with disabilities so that they are able to play a full and active role in society. The goal is to guarantee solid steps that are taken for equality in the enjoyment of fundamental rights and responsibilities on an equal basis just like other Malawian citizens. To this effect, the policy concedes disability as a social construct and aims at giving guidelines for the inclusion of disability issues in the Government's development agenda as a human rights issue. It calls for combined efforts and harmonized management systems for planning, implementation, and monitoring at all levels.

One of the priority areas, as pointed out in section 4.12, is assistive technologies. Section 4.6.1.2^34^ of the National Policy on Equalization of Opportunities for Persons with Disabilities states the need to “design and develop appropriate technologies, assistive devices, and learning materials.” Thus, a Policy Framework at the national level for appropriate technology in Malawi is available. “The objective of this priority area is to promote and support disability research and the development and application of appropriate technologies for disability programmes”^35^. However, there seems to be little on the ground.

The section includes strategies that would be used to achieve the above that includes the availability of financial and technical assistance to key stakeholders; boosting innovations in appropriate technologies; facilitate coordination in disaggregated data collection and research in all relevant studies including census. It also includes dissemination of data to all planners and stakeholders in the disability programming. Establishment of a national user-friendly Disability Management Information System (DMIS) interfaced with other management information systems in various sectors, for example, health, education, among others are part of the strategy currently being done.

#### Education Policy and Investment Framework (PIF)^38^ and the National Education Sector Policy, 2016^39^

Inclusive primary education is the goal of the Education Sector Investment Framework as well as the national education policy. The policies are aimed at developing and managing an education system that would increase enrolment, retention, and graduation of children with disabilities from school. It then offers improved access to the school environment that includes assistive technologies. Following these policy agenda, the Government has launched an Inclusive Education Strategy^40^ that also targets learners with intellectual disabilities and could assist them to access assistive products in order to equalize their education opportunities.

#### TEVETA Policy and TEVET Act (2nd Edition of 2013)

The Technical Entrepreneurial, Vocational Education and Training Authority (TEVETA) through its TEVET Act of 1999^41^, Policy^42^, and Strategic Plan^43^ prioritize persons with disabilities and other vulnerable persons as a fundamental constituent of the authority. Under the pillar “Access and Equity” the strategic plan^44^ supports access to vocational skills training by persons with disabilities.

#### Malawi National Health Policy, 2012^45^

The healthy policy emphasizes the inclusion of the vulnerable groups that include persons with disabilities, women and the elderly in all health interventions. It is key to access of persons with intellectual disabilities to assistive technologies since the element of assistive technologies, in question, falls under the WHO CBR Guidelines Health Component.^46^

## Recent Developments

The most recent development in Assistive Technology that Malawi has experienced was attending the summit on Assistive Technology in Geneva Switzerland from the 3rd to 4th August 2017. The summit was initiating a “Global Priority Research Agenda for Improving Access to High-Quality Affordable Assistive Technologies and Devices.” In short, it is called the GREAT Summit. Following the summit, a report was submitted to the Principal Secretary for the Ministry of Gender, Children, Disability and Social Welfare so that action is taken in Malawi so that it can move together with the rest of the world.

This prompted during the drafting of the Disability Bill 2019 for Appropriate Technologies to be included^47^. The Bill includes broader strategies for appropriate technology in Malawi to developing sectoral policies and legislation on health in line with the National Policy on Equalization of Opportunities for Persons with Disabilities (2006) article 4.12^48^ and the Disability Act 2012 section 26^49^.

The other broader strategy is strengthening networking among public and private service providers in the areas of promotive, preventive, medical, and rehabilitative services to ensure a more coordinated case management. A good example under CBID is the Community Training on making and repairing of simple assistive devices (e.g., Appropriate Paper Technology -APT)^50^.

## Actionable Recommendations

Despite the numerous policies and legislation that would enable persons with intellectual disabilities to access assistive technology, there is though little happening on the ground ^51^. There are numerous challenges like level of access and quality of the services for persons with disabilities for the past years; lack of data and statistics in the Malawi Growth and Development Strategy and previous absence of a Healthy Policy and legislation; limited access to mobility and other devices due to no decentralization of rehabilitation services within the Ministry of Health delivery structure; and inadequate number of specialists such as orthopedic surgeons, ophthalmologists, physiotherapists, and occupational therapists, rehabilitation technicians, medical social workers, and community nurses^52^.

Nonetheless, the Community Based Inclusive Development (CBID) promises to be a solution to the problem. CBID is a rights-based approach that is focused on development with an aim to mainstream disability in development work and in the Governments development agenda. Dissemination of appropriate technology information is already taking place through CBID. Therefore, there are features that would make CBID a recommended tool to remove the challenges of intellectual disabilities and access to appropriate technology.

The features of CBID that would assist this cause are diversification and inclusiveness that give answers to a wide and specific social-economic requirements of persons with varied types of disabilities, age groups and different cultural contexts; partnership and collaboration that permit sharing of best practices and accountabilities as well as gives a wide-ranging service delivery. Features also include the capacity building that consists of the transfer of skills for mainstreaming disability in development work from the grass-roots up to the national level; participation of persons with disabilities in CBID that calls for their active involvement and participation as rights holders in the spirit of “Nothing about Us without Us.”

Data collection and management, which gives effective planning, implementation, monitoring and evaluation of the programme is an important aspect hence Malawi has come up with a National Disability Monitoring and Evaluation framework, Inclusive Education Strategy and the National Mainstreaming Strategy. The National Coordination and Consultative Committee on Disability Issues

(NACCODI) has also been re-instituted by the Honorable Minister of Gender, Children, Disability and Social Welfare. This is a committee of Principal Secretaries to ensure that there is coordination among all Government sectors so that mainstreaming of disability is done.

Enhancing international cooperation as per the United Nations Convention on the Rights of Persons with Disabilities would be very important. For example, after the GREAT Summit in Geneva, the African Commission considered the possibility of concerted efforts to address issues of appropriate Technology in Africa^53^. Similarly, a provisional position paper was drafted at the University of Southern Queensland in the United Kingdom toward an international framework to ensure the availability and accessibility of affordable high-quality assistive technologies globally.

The provisional paper highlighted the need for states to develop national policies on appropriate technology provision that includes a policy on appropriate technology manufacturing; use of appropriate technology mainstreaming technologies to intensely stimulate appropriate technology and services; ensure evidence-based information on appropriate technology and allied services available. On top of that develop training programmes in appropriate technology like has been done in Malawi in the development of training packages for CBID that has included appropriate technology.

Malawi has picked the cue since the issue of appropriate technology was also discussed with the Principal Secretary for the Ministry of Gender, Children, Disability and Social Welfare in a meeting on the 25th of October, 2018 at Mount Soche Hotel in Blantyre, Malawi, where the Principal Secretary implored organizations like the Ministry itself, the Malawi Council for the Handicapped and the Federation of Disability Organizations in Malawi to greatly include appropriate technology in their programmes and services.

## Conclusion

The introduction of the new harmonized CBID model in Malawi that matches the principle of the Sustainable Development Goals to “**Leave No One Behind**” is a great milestone. There is now hope that the aspect of intellectual disability and access to assistive technologies will improve further using the Mainstreaming Strategy, the CBID and other informal community structures. NACCODI will also play an important role to ensure that nobody is segregated from realizing his or her rights.

There is, nonetheless, need for reinforcement of legal policy instruments that are already in place and systemic reforms to ensure inclusive programming and development to achieve inclusivity of persons with intellectual disability.

Progressively, the challenges may be declining but it needs concerted efforts from all key partners and the much-needed coordination from the CBID programme.

## Author Contributions

The author confirms being the sole contributor of this work and has approved it for publication.

### Conflict of Interest Statement

The author declares that the research was conducted in the absence of any commercial or financial relationships that could be construed as a potential conflict of interest.

## Footnotes

^1^Disability in Uganda: Focus on Tororo District intellectual disability in Malawi may also consist of mental illness. http://www.jldd.jp/bestpractice/BP2008(Uganda).pdf (As of 25 October 2018).

^2^National Policy on Equalization of Opportunities for Persons with Disabilities (Malawi Government Print, 2006) www.nyasatimes.com/malawi-on-national-inclusive-education-strategy/ (As of 7 November 2018).

^3^Previously known as Community Based Rehabilitation (CBR).

^4^The Malawi Growth and Development Strategy (MGDS III). https://cepa.rmportal.net/Library/government-publications/the-malawi-growth-and-developmentstrategy-mgds-iii (As of 25 October 2018).

^5^WHO CBR Matrix www.who.int/disabilities/cbr/matrix/en/ (As of 7 November 2018).

^6^The National Mainstreaming Strategy http://www.maravipost.com/malawigovernment-commitson-disabilities-rights-launches-mainstreaming-strategyand-implementation-plan/ (As at 7 November 2018).

^7^Disability Act (Malawi Government Print, 2012) www.scotlandmalawipartnership.org/download_file/267/207 (As of 7 November 2018).

^8^UN Enable, “Conventional and Optional Protocol Signatures and Ratifications” http://www.un.org/disabilities/documents/convention/convoptprot-e.pdf (As of 6 March 2018).

^9^See National Disability Policy (n 2 above).

^10^See National Disability policy (n 2 above).

^11^SINTEF is an independent research institute https://www.sintef.no/en/this-is-sintef/ (As of 25 October 2018). It has not been given in full.

^12^Loeb and Eide, “Living Conditions among People with Disabilities in Malawi,” http://www.washingtongroup-disability.com/wpcontent/uploads/2016/03/SINTEF_malawi_report.pdf. (As of 23 March 2018).

^13^Interview with Lucy Magagula – Acting Principal, Montfort Special Needs Education College (Lilongwe, Malawi, 8 February 2018).

^14^Interview with Enoch Mithi – Programme Manager, Parents of Disabled Children Association of Malawi (Liwonde, Malawi, 23 February 2018).

^15^See (n 1 above).

^16^Assistive Devices and Equipment, Disability Info SA uhambofoundation.org.za/new-wpcontent/upload/2016/… (As of 25 October 2018).

^17^As above.

^18^See Disability in Uganda (n 1 above).

^19^Convention on the Rights of Persons with Disabilities (United Nations, 2006) https://www.un.org/development/desa/disabilities/convention-on-the… (As of 7 November 2018).

^20^(As above) art. 4g and 4h.

^21^(As above) art. 20 b and 20 d.

^22^(As above) art. 26(3).

^23^(As above) art. 29a (ii).

^24^(As above) art 32d.

^25^The Constitution of the Republic of Malawi Act 20 of 1994 https://www.wipo.int/edocs/lexdocs/laws/en/mw/mw030en.pdf (As of 6 March 2018).

^26^(As above) sect. 13.

^27^(As above) sect. 13g.

^28^See the Disability Act http://www.fedomamalawi.org/public/themes/Fedoma/downloads/Disability_Act_2012.pdf (n 9 above).

^29^See the National Disability Policy (n 2 above) sect 4.6.1.2.

^30^The Malawi Education Act https://www.malawilii.org/…/legislation/…act/…/num_act_2012_35. (As of March 2018).

^31^The Malawi Handicapped Persons Act 1971 https://dredf.org/legal-advocacy/international-disability-rights/international-laws/malawi-handicapped-personsact/. (As of 23 March 2018).

^32^The Malawi Employment and Labour Act 2000 http://www.intosa.com/uploads/download/file/289/Employment_Act__2000_.pd. (As of 23 March 2018).

^33^See National Disability Policy (n 2 above).

^34^(As above) sect. 4.6.1.2.

^35^(As above) sect 4.12.

^38^Malawi Education Policy and Investment Framework (PIF) http://www.sdnp.org.mw/Education2010/Edu-policy.html. (As of March 23, 2018).

^39^(As above).

^40^Malawi Inclusive Education Strategy https://www.nyasatimes.com/malawi-on-national-inclusiveeducation… (As of March 23, 2018).

^41^TEVET Act 1999, https://www.google.com/search?q=TEVET+Act+malawi&oq=TEVET+Act+malawi&aqs=chrome.69i57.14053j0j4&sourceid=chrome&ie=UTF-8.php/downloads/Tevet-act-pdf/detail (As of March 23, 2018).

^42^TEVET Revised Policy 2013, https://www.google.com/search?ei=KEY0XNuIN8XgUazJmLAL&q=TEVET+policy+malawi&oq=TEVET+policy+malawi&gs_l=psy-ab.12…164389.165964.168275…0.0.0.1945.1945.8-1……0….1.gws-wiz.7DSvzXOfs_M Revised policy final.pdf/download (As of March 23, 2018).

^43^TEVETA Strategic Plan 2013-2018, http://www.teveta.mw/index.php/downloads/TEVETA Strategic Plan 2013 to 2018 (As of March 23, 2018).

^44^TEVETA Strategic Plan (n 44 above) 16.

^45^Malawi National Health Policy https://www.google.com/search?q=national+health+policy+malawi&oq=national+health+policy+malawi&aqs=chrome.69i57.14667j0j4&sourceid=chrome&ie=UTF-8 (As of March 23, 2018).

^46^WHO CBR Guidelines Health - Component https://www.ncbi.nlm.nih.gov/books/NBK310926/ (As of March 23, 2018).

^47^The Malawi Disability Bill 2018, Sect, 16.

^48^See the National Disability Policy (n 2 above).

^49^See the Disability Act (n 9 above).

^50^See (n 21 above).

^51^See National Disability Policy (n 2 above).

^52^See National Mainstreaming Strategy https://www.nyasatimes.com/ministerkalirani-condemns-exploitation-of-people-with-disabilities-malawi-launchesmainstreaming-strategy/ (n 9 above).

^53^A letter was written by Lefhoko of the African Commission to come up with a continental effort to address the challenge of Assistive Technologies in Africa by coming up with guidelines, standards, policy and a strategy.
